# ZIP10 is a negative determinant for anti-tumor effect of mannose in thyroid cancer by activating phosphate mannose isomerase

**DOI:** 10.1186/s13046-021-02195-z

**Published:** 2021-12-09

**Authors:** Sharui Ma, Na Wang, Rui Liu, Rui Zhang, Hui Dang, Yubo Wang, Simeng Wang, Zekun Zeng, Meiju Ji, Peng Hou

**Affiliations:** 1 Department of Endocrinology, The First Affiliated Hospital of Xi’an Jiaotong University, Xi’an, 710061 People’s Republic of China; 2 Department of Endocrinology, Xi’an Central Hospital, Xi’an, 710003 People’s Republic of China; 3 Department of Radio-Oncology, The First Affiliated Hospital of Xi’an Jiaotong University, Xi’an, 710061 People’s Republic of China; 4 Center for Translational Medicine, The First Affiliated Hospital of Xi’an Jiaotong University, Xi’an, 710061 People’s Republic of China

**Keywords:** Thyroid cancer, Mannose, Phosphate mannose isomerase (PMI), ZIP10, Cellular glycolysis

## Abstract

**Background:**

Mannose, a natural hexose existing in daily food, has been demonstrated to preferentially inhibit the progression of tumors with low expression of phosphate mannose isomerase (PMI). However, its function in thyroid cancer still remains elusive.

**Methods:**

MTT, colony formation and flow cytometry assays were performed to determine the response of thyroid cancer cells to mannose. Meanwhile, mouse models of subcutaneous xenograft and primary papillary thyroid cancer were established to determine in vivo anti-tumor activity of mannose. The underlying mechanism of mannose selectively killing thyroid cancer cells was clarified by a series of molecular and biochemical experiments.

**Results:**

Our data demonstrated that mannose selectively suppressed the growth of thyroid cancer cells, and found that enzyme activity of PMI rather than its protein expression was negatively associated with the response of thyroid cancer cells to mannose. Besides, our data showed that zinc ion (Zn^2+^) chelator TPEN clearly increased the response of mannose-insensitive cells to mannose by inhibiting enzyme activity of PMI, while Zn^2+^ supplement could effectively reverse this effect. Further studies found that the expression of zinc transport protein ZIP10, which transport Zn^2+^ from extracellular area into cells, was negatively related to the response of thyroid cancer cells to mannose. Knocking down ZIP10 in mannose-insensitive cells significantly inhibited in vitro and in vivo growth of these cells by decreasing intracellular Zn^2+^ concentration and enzyme activity of PMI. Moreover, ectopic expression of ZIP10 in mannose-sensitive cells decrease their cellular response to mannose. Mechanistically, mannose exerted its anti-tumor effect by inhibiting cellular glycolysis; however, this effect was highly dependent on expression status of ZIP10.

**Conclusion:**

The present study demonstrate that mannose selectively kills thyroid cancer cells dependent on enzyme activity of PMI rather than its expression, and provide a mechanistic rationale for exploring clinical use of mannose in thyroid cancer therapy.

**Supplementary Information:**

The online version contains supplementary material available at 10.1186/s13046-021-02195-z.

## Background

The incidence of thyroid cancer has augmented dramatically worldwide [1, 2], and it recently ranks third of all malignant tumors among Chinese women [3]. Thyroid cancers are classified into differentiated and undifferentiated thyroid cancers [4]. The former accounts for the vast majority of thyroid cancers, which can be efficiently cured by thyroidectomy combined with postoperative thyroid hormone suppression and radioactive iodine treatment [5]. However, there are still minority patients who relapse and develop into undifferentiated thyroid cancer, which are resistant to most conventional therapy, resulting in poor prognosis and fatal outcomes [6]. Thus, there is an urgent need to develop effective and safe therapy for this disease.

Mannose, a natural hexose existing in daily food, takes part in several physiological processes, such as energy metabolism, protein glycosylation and immune reaction [7–9]. As mannose is not detrimental to human health, some studies have explored its clinical function as a drug. For example, mannose has been demonstrated to treat urinary tract infections, especially in female patients who suffer from recurrent infections [10, 11]. There is also study showing that mannose can alleviate the progression of type 1 diabetes by activating Treg cells and suppressing immunity [12, 13]. Besides, a previous study proved that mannose could change the intestinal flora, thereby improving energy metabolism. Thus, it is considered as a potential weight-loss medicine [14]. In recent years, more attention has been paid to its antitumor role. For example, a previous study showed that higher concentration of plasma mannose predicted better prognosis for patients with esophageal adenocarcinoma [15]. It has been revealed that mannose preferentially killed cancer cells which expressed lower phosphate mannose isomerase (PMI) [16].

PMI is ubiquitous in prokaryotic and eukaryotic cells as a housekeeping enzyme, which reversibly catalyze the conversion of mannose-6-phosphate (M-6-P) and fructose- 6-phosphate (F-6-P) to participate in energy metabolism [17]. Mannose is transported into tumor cells through glucose transporter GLUTs and converts into M-6-P by hexokinase. When PMI is lowly expressed in tumor cells, M-6-P cannot be transformed into F-6-P, leading to accumulation of M-6-P and inhibition of glycolysis. In this way, the response of tumor cells to mannose is related to PMI protein expression [16]. It is clear that the function of PMI relies on zinc ions (Zn^2+^) which participate in the structure and activity of several proteins [17, 18].

The metal ion transporter families SLC39 and SLC30 regulate import and export of Zn^2+^ to meet steady state of cell environment. The SLC30 family (ZnT family) contains ten members which are responsible for transporting Zn^2+^ from the cytoplasm to extracellular area or organelles, while the SLC39 family (alias ZIP family) consists of 14 members that shift Zn^2+^ from extracellular area or organelles to the cytoplasm [19, 20]. ZIP10, an important member of the SLC39 family, exerts various physiological and pathological functions by affecting Zn^2+^ concentration and changing enzyme activity. For example, there is evidence showing that ZIP10 influences histone acetyltransferase by controlling intracellular Zn^2+^ concentration, keeping skin healthy [21]. Meanwhile, it has been proved that ZIP10 knockdown induces apoptosis in early B-cell as ZIP10 regulates caspase activity by Zn^2+^ balance [22].

In this study, our data showed that mannose could selectively kill thyroid cancer cells by a series of in vitro and in vivo studies, and this effect was highly dependent on ZIP10 expression levels, but not PMI expression levels.

## Materials and methods

### Cell culture and drug treatment

Human thyroid cancer cell lines TPC-1, BCPAP, FTC133, IHH4, 8305C, 8505C and K1 were kindly provided by Dr. Haixia Guan (Guangdong Provincial People’s Hospital, Guangzhou, P.R. China). These cell lines were authenticated by analyzing short tandem repeat at Genesky Co. Ltd., (Shanghai, P.R. China), and the results (Additional file 1: Table S1) were consistent with a previous study [23]. We routinely cultured these cells at 37 °C in RPMI-1640 (Gibco) or DMEM/Ham’s F-12 (Gibco) medium with 10% fetal bovine serum (FBS), and treated cells with D-Mannose (Aladdin) or TPEN (Sigma) at the indicated concentrations and time points.

### Cell viability and colony formation assays

Cells (800 to 2000/well) were seeded in 96-well plates. After cell attachment to the plate, different doses of D-mannose were added into culture medium at the time points. Next, we performed the MTT assay to determine the effect of mannose on cell proliferation, and then calculate IC_50_ value of each cell line as described previously [24].

Cells (3000 to 5000/well) were seeded on 12-well plates and cultured with medium containing gradually increasing concentrations of D-mannose for 7 days. After cells were fixed, washed and stained, colony number was counted under an inverted microscope. We defined more than 50 cells as a colony. Each assay was carried out in triplicate.

### Cell cycle assay

After attachment to cell plate, cells were cultured in serum-free medium for 12 h and treated with mannose or not for 24 h. Next, we fixed cells with 66% cold methanol for at least 2 h. Cells were then stained with PI and subjected to flow cytometry to analyze cell cycle distributions.

### Short interfering RNAs (siRNAs), lentivirus short hairpin RNAs (shRNAs) and expression plasmids

Oligonucleotides of siRNAs targeting PMI or ZIP10 and control siRNAs were purchased from Ruibobio (Guangzhou, China), and the sequences were showed in Additional file 1: Table S2. One day before transfection, cells were seeded on a 12-well plate to achieve 50% confluence overnight. Next, we transfected these cells with the above siRNAs at a final concentration of 50 nM using X-treme GENE siRNA Transfection Reagent (Roche Diagnostics).

Lentivirus encoding PMI-shRNA, ZIP10-shRNA, control shRNA, PHBLV-ZIP10 and PHBLV-vector were purchased from HanBio Biotechnology Co., Ltd. The sequences of shRNAs were shown in Additional file 1: Table S3. Cells were cultured to achieve 50% confluence and transfected with different constructs or a final lentivirus multiplicity of infection (MOI) of 10–100.

### RNA extraction and quantitative RT-PCR (qRT-PCR)

RNA extraction, cDNA synthesis and qRT-PCR assays were performed as described previously [25]. The mRNA expression was normalized to *18S* rRNA. The primer sequences were shown in Additional file 1: Table S4. Each sample was analyzed in triplicate.

### Western blot analysis

Cells were cultured and treated with the indicated conditions. After cells were washed and lysed, equal amounts of protein lysates were subjected to 10% SDS-PAGE electrophoresis, and transferred onto polyvinylidene fluoride membranes (Roche Diagnostics GmbH, Mannheim, Germany). Next, we incubated the membranes with primary antibodies at 4 °C overnight as follow: anti-PMI (Abcam), anti-ZIP10 (Novus Biologicals), anti-cyclin D (Abcam), anti-cyclin E (Santa Cruz), anti-phospho-CDK2 (pCDK2, CST), anti-CDK2 (ABclonal), anti-p53(Santa Cruz), and anti-β-actin (Abcam). After being immunoblotted with corresponding secondary antibodies, immunoblotting signals were collected using the Western Bright ECL detection system (Advansta, Menlo Park, CA).

### Measurement of PMI enzyme activity

Cysteine carbazole sulfuric acid method was used to measure enzyme activity of PMI as described previously [26]. In brief, cells were washed, and then lysed by three freeze–thaw cycles and Ultrasonic cracker on ice. Next, the reactions were initiated by the addition of equal protein samples into the reaction buffer containing 40 mM Tris-HCl pH 7.4, 6 mM MgCl_2_, 5 mM Na_2_HPO_4_/KH_2_PO_4_ and 20 mM mannose-6-phosphate. After a 2-h incubation, the reactions were stopped by adding 1.5% cysteine hydrochloride and 0.12% alcoholic solution of carbazole with concentrated sulfuric acid. After shaking this reaction buffer, the amount of complex formed was estimated spectrophotometrically in a spectrophotometer at 560 nm at room temperature. In parallel, β-actin was detected by western blot analysis to prove protein amount consistency.

### Measurement of intracellular Zn^2+^

Cells were seeded in a 6-well plate and treated with TPEN or the indicated siRNAs. After a 48 h-treatment, cells were washed and incubated with FluoZin™-3 (Invitrogen) at concentration of 1 μM for 30 min in a dark 37 °C carbon dioxide incubator. Next, we washed and incubated cells with PBS at 37 °C for 20 min in a carbon dioxide incubator to de-esterify. The concentration of intracellular Zn^2+^ was then measured by flow cytometer.

### Animal studies

Eight-week-old female BALB/c athymic mice were purchased from SLAC laboratory Animal Co., Ltd. (Shanghai, China). Next, we subcutaneously injected 8305C/sh-NC cells (1 × 10^7^), 8305C/sh-ZIP10 cells (1 × 10^7^) and FTC133 cells (1 × 10^7^) into armpit region of these mice to establish xenograft mouse model, and randomly divided them into two groups (vehicle control vs. mannose; five mice/group). Normal water was then replaced by 15% mannose for mannose group. Meanwhile, mice received 20% mannose water or sterilized water by oral gavage (150 μL) four times per week. We weighted mice every 2 days, measured length and width of tumors using digital caliper, and calculated tumor volumes according to the formula: length × width^2^ × 0.5. In the end of the experiments, the mice were given final oral gavage of mannose and sacrificed 5 h later. Tumors were gathered and weighted. Part of xenograft tumors was used for western blot analysis, while the others were used for IHC assay. The antibody used for IHC assays were as follows: Ki-67 (Abcam), cyclin D (Abcam), cyclin E (Santa Cruz) and pCDK2 (ABclonal). Image J software was used to analyze the staining levels of Ki-67, cyclin D, cyclin E and pCDK2. The staining point was then scored 0, 1, 2, 3 representing negative, weak positive, positive and strong positive. Next, we quantified staining levels using the score multiply relating proportion and plus together. The staining levels of each sample was presented relative to the deepest staining of control group.

*TPO-Cre* and *Braf*^*CA*^ mice were kindly provided by Drs. Kimura Shioko (National Institutes of Health, USA) and Martin McMahon (University of California, USA), respectively. These two strains of mice mated and produced mice with *Braf*^*V600E*^-driven thyroid cancer according to a previous study [27]. Mice were randomly divided into two groups, and then received 20% mannose water or sterilized water by oral gavage (200 μL) every day, respectively. Next, we detected tumor burden by Vevo1100 ultrasound imaging system every week. The area of largest thyroid cross-section was quantified and normalized to the size at week 4 as described previously [28]. At the end of the experiments, all mice were sacrificed, and thyroids were collected and weighted. The tissues were then fixed, embedded and sectioned for IHC assays. The above experiments were approved by the Laboratory Animal Center of Xi’an Jiaotong University.

### Seahorse glycolytic stress test

The extracellular acidification rate (ECAR) which indicates for glycolysis function was measured by using seahorse XF glycolysis stress kit and XF96 analyzer (seahorse biosciences) as described previously [29]. First, cells (20000–40,000 cells/well) were seeded in seahorse XF96 microplates. After attachment to the plate, cells were treated with 20 mM D-mannose or not for 36 h, washed twice with base medium (seahorse bioscience) containing 2 mM glutamate, and incubated in a non-CO_2_ incubator for 1 h. after calibration, a final concentration of 10 mM glucose, 1 mM oligomycin, and 50 mM 2-deoxy-D-glucose were injected sequentially into cells. Changes in extracellular acid rate (ECAR) after the injection of glucose (activating glycolysis), oligomycin (suppressing mitochondrial adenosine triphosphate synthase) and 2-deoxy-D-glucose (suppressing glycolysis) represent cellular glycolysis and its maximum capacity, respectively.

### Statistical analysis

All data were analyzed using GraphPad Prism 8.3.0 software. Reed-Muench method was used to calculate IC_50_ values. Student’s *t*-tests were used to compare the control and treatment groups. Data were represented as mean ± SD. A difference of *P* < 0.05 indicated statistical significance.

## Results

### Mannose selectively inhibits the growth of thyroid cancer cells in vitro

**Fig. 1:**
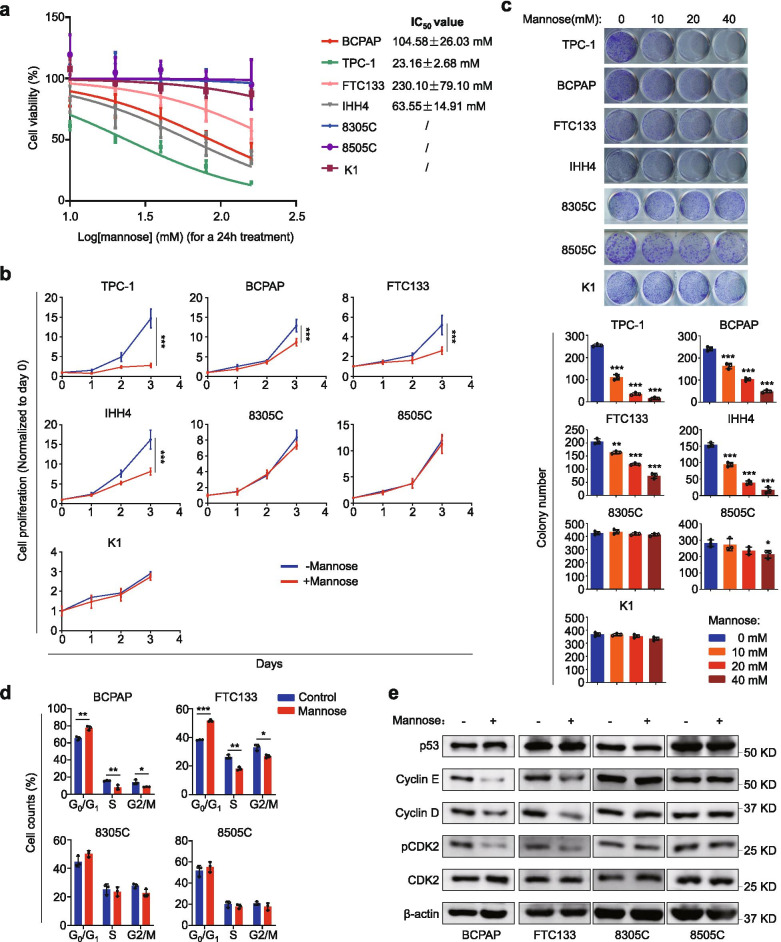
Selective inhibition of thyroid cancer cell growth by mannose. **a** Thyroid cancer cell lines TPC-1, BCPAP, FTC133, IHH4, 8305C, 8505C and K1 were treated with mannose in dose-dependent manner for 24 h. Cell viability was then evaluated by MTT assay, and IC_50_ values were calculated using the Reed-Muench method. **b** The above cells were treated with 20 mM mannose or not for 3 days. MTT assays were then performed to assess the proliferation of these cells in time-dependent manner. **c** Colony formation assay was performed when these cells were treated with mannose in dose-dependent manner for 7 days (upper panels). Quantitative analysis of colony numbers was presented in the lower panels. **d** Cells were treated with mannose or not for 24 h, and stained with PI. Cell cycle distributions were then analyzed by the flow cytometry. **e,** Western blot analysis of cell cycle-related molecules (including p53, cyclin E and cyclin D, pCDK2 and CDK2) in the indicated cells with a 24-h mannose treatment or not. β-actin was used as a loading control. The data were presented as mean ± SD. Statistically significant differences were indicated: *, *P* < 0.05; **, *P* < 0.01; ***, *P* < 0.001

To determine the effect of mannose on the proliferation of thyroid cancer cells, we treated seven human thyroid cancer cell lines with increasing concentration of mannose from 20 mM to 160 mM for 24 h, and calculated their IC_50_ values using MTT assays. As shown in Fig. 1a, TPC-1, BCPAP, FTC133 and IHH4 cells were more sensitive to mannose, while 8305C, 8505C and K1 cells were not sensitive to mannose. Next, we evaluated time-dependent response of these cell lines to mannose by treating them with 20 mM mannose. Similarly, mannose significantly suppressed the proliferation of TPC-1, BCPAP, FTC133 and IHH4 cells in a time-dependent manner, while almost did not affect the proliferation of 8305C, 8505C and K1 cells (Fig. 1b). Besides, to determine whether mannose affected the proliferation of normal thyroid cells, we treated the immortalized thyroid cells Hthy-ori3–1 with 20 mM mannose. The results showed that the proliferation of Hthy-ori3–1 cells was hardly affected by mannose (Additional file 2: Fig. S1), suggesting that mannose is a safe agent for the treatment of thyroid cancer.

Next, we performed colony formation assays to confirm anti-tumor ability of mannose in thyroid cancer cells by treating the above cell lines with different doses of mannose for 7 days. As expected, mannose significantly inhibited colony formation ability of TPC-1, BCPAP, FTC133 and IHH4 cells in a dose-dependent manner, while barely affected colony formation of 8305C, 8505C and K1 cells (Fig. 1c). Besides, we also assessed the effect of mannose on cell cycle distributions of thyroid cancer cells. The results showed that mannose caused a significant G_0_/G_1_ phase arrest in BCPAP and FTC133 cells, while remained almost unchanged in 8305C and 8505C (Fig. 1d and Additional file 2: Fig. S2a). In the cell cycle regulation of cancer cells, cyclin D determined early entry in G_1_ phase and then cyclin E regulated transition from G_1_ to S [30, 31]. Cyclin E interacted with CDK2 and phosphorylated CDK2 (pCDK2), triggering cell cycle progression [32]. Moreover, p53 as an important tumor suppressor induces G_0_/G_1_ phase arrest [33]. Thus, we investigated the effect of mannose on these molecules. The results showed that mannose did not change p53 expression in both mannose-sensitive and -insensitive cells. However, mannose clearly inhibited the expression of cyclin D, cyclin E and pCDK2 in mannose-sensitive cell lines BCPAP and FTC133, but not in mannose-insensitive cell lines 8305C and 8505C (Fig. 1e and Additional file 2: Fig. S2b). Collectively, our data demonstrate that mannose can selectively inhibit the growth of thyroid cancer cells.

### PMI knockdown increases the response of mannose-insensitive thyroid cancer cells to mannose

**Fig. 2:**
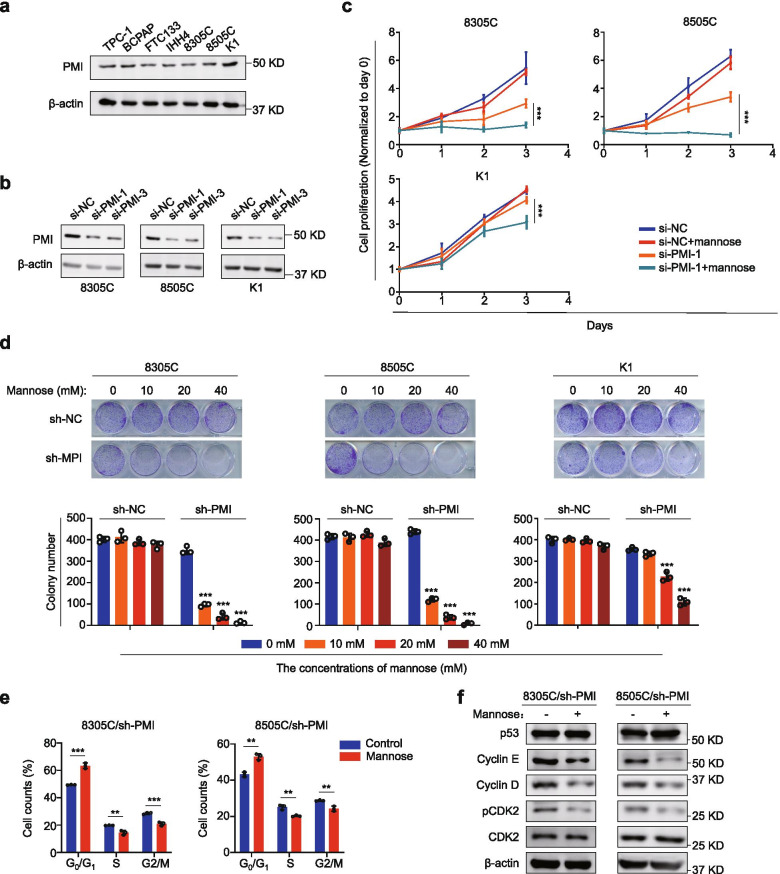
PMI knockdown improves mannose-insensitive thyroid cancer cell responsiveness to mannose. **a** Western blot analysis of PMI in thyroid cancer cell lines. β-actin was used as a loading control. **b** Western blot analysis showing PMI knockdown in 8305C, 8505C and K1 cells by two different siRNAs (si-PMI-1 and si-PMI-3). β-actin was used as a loading control. **c** MTT assays showing the proliferation of the indicated cells treated with 20 mM mannose and control cells. **d** PMI knockdown-8305C, 8505C and K1 cells, and control cells were treated with mannose in a dose-dependent manner. The representative colony images were shown in the upper panels. Colony numbers were then counted and presented in the lower panels. **e** PMI knockdown-8305C and 8505C cells were treated with mannose or not for 24 h, and stained with PI. Cell cycle distributions were then analyzed by the flow cytometry. **f** Western blot analysis of cell cycle-related molecules (including p53, cyclin E and cyclin D, pCDK2 and CDK2) in PMI knockdown-8305C and 8505C after a 24-h mannose treatment. β-actin was used as a loading control. The data were presented as mean ± SD. Statistically significant differences were indicated: **, *P* < 0.01; ***, *P* < 0.001

As reported previously, the response of cancer cells to mannose related to expression levels of phosphate mannose isomerase (PMI) protein [16]. First, we assessed the expression of PMI in these thyroid cancer cells by western blot analysis. However, we did not find significant difference in PMI expression among them (Fig. 2a and Additional file 2: Fig. S3a). Next, we knocked down PMI using two different PMI siRNAs in mannose-insensitive cell lines 8305C, 8505C and K1 (Fig. 2b and Additional file 2: Fig. S3b), and found that PMI depletion significantly enhanced the response of these cells to mannose compared to the control (Fig. 2c). To further verify the above conclusion, we stably knocked down PMI in 8305C, 8505C and K1 cells using lentivirus system (Additional file 2: Fig. S3c), and expectedly found that mannose inhibited colony formation of these cells in a dose-dependent manner, but not in control cells (Fig. 2d). Similarly, we also determined the effect of mannose on cell cycle distributions in PMI knockdown-8305C and 8505C cells. The results showed that mannose significantly induced G_0_/G_1_ phase arrest of these cells (Fig. 2e and Additional file 2: Fig. S3d), and inhibited the expression of cyclin D, cyclin E and pCDK2, while did not change p53 expression (Fig. 2f and Additional file 2: Fig. S3e). These data indicate that PMI depletion enhances the response of mannose-insensitive thyroid cancer cells to mannose.

### Intracellular Zn^2+^ concentration affects PMI activity and the response of thyroid cancer cells to mannose

**Fig. 3:**
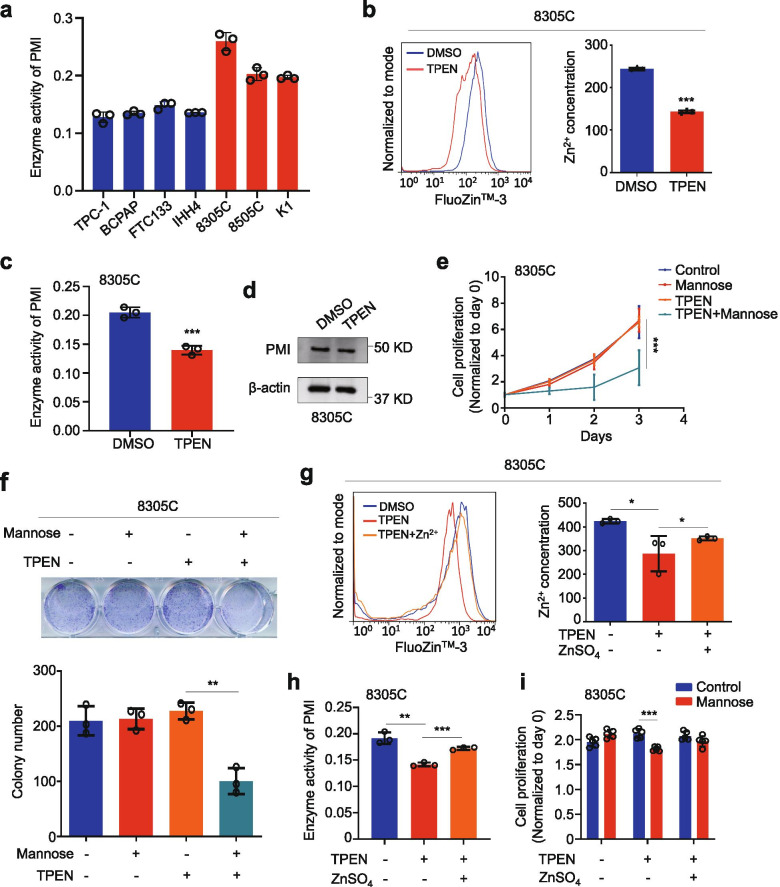
The effect of intracellular Zn^2+^ concentration on enzyme activity of PMI and thyroid cancer cell responsiveness to mannose. **a** Enzyme activity of PMI in thyroid cancer cells was examined by cysteine carbazole sulfuric acid method. The O.D. values were measured in a spectrophotometer at 560 nm. **b** The fluorescence was analyzed to measure intracellular Zn^2+^ concentration after TPEN treatment and FluoZin™-3 incubation by flow cytometer (left panel). Statistical analysis of fluorescence intensity by Student’s t test (right panel). **c** Enzyme activity of PMI was measured by cysteine carbazole sulfuric acid method after TPEN treatment. **d** Western blot analysis of PMI in TPEN or DMSO-treated 8305C cells. β-actin was used as a loading control. **e** The MTT assay showing the effect on mannose on the proliferation in 8305C cells with different treatments. ***, *P* < 0.001 for TPEN vs. TPEN + mannose. **f** The effect of mannose on colony formation in 8305C cells with different treatments (upper panel). Colony numbers were counted and presented in the lower panel. **g** Measurement of intracellular Zn^2+^ concentration in 8305C cells with the indicated treatments by flow cytometer (left panel). Statistical analysis of fluorescence intensity by Student’s t test (right panel). **h** Enzyme activity of PMI was tested in 8305C cells with the indicated treatments. The O.D. values were measured in a spectrophotometer at 560 nm. **i** The MTT assay showing the effect of mannose on the proliferation of 8305C cells with the indicated treatments. The data were presented as mean ± SD. Statistically significant differences were indicated: *, *P* < 0.05; **, *P* < 0.01; ***, *P* < 0.001

Considering that, in addition to gene/protein expression, enzyme activity can also affect their function and cellular response to drug treatment [34]. Cysteine carbazole sulfuric acid method was usually used to test enzyme activity of PMI [26]. First, we found that enzyme activity of PMI in 8305C and 8505C cells decreased when PMI was knocked down (Additional file 2: Fig. S4), proving the accuracy of this method. Next, we tested enzyme activity of PMI in all mannose-sensitive and -insensitive cells using this method. The results showed that enzyme activity of PMI was clearly lower in mannose-sensitive cell lines than mannose-insensitive cell lines (Fig. 3a).

It is clear that PMI depends on Zn^2+^ to exert its enzymatic function [17]. To prove whether intracellular Zn^2+^ concentration affects the response of cancer cells to mannose, we treated 8305C cells with 3.5 μM Zn^2+^ chelator TPEN, and demonstrated that TPEN treatment significantly decreased intracellular Zn^2+^ concentration compared to the control (Fig. 3b). Meanwhile, we expectedly found that PMI enzyme activity was significantly reduced upon TPEN treatment (Fig. 3c); however, TPEN treatment did not affect PMI expression compared to the control (Fig. 3d). Besides, we found that TPEN treatment dramatically increased the response of mannose-insensitive cell line 8305C to mannose (Fig. 3e). This was also supported by the results of colony formation assays (Fig. 3f and Additional file 2: Fig. S5). Next, we demonstrated that Zn^2+^ concentration and enzyme activity of PMI could be partially recovered by supplementing 5 μM ZnSO_4_ in culture medium of 8305C cells after TPEN treatment (Fig. 3g and h). As expected, we found that the sensitizing effect of TPEN on the response of 8305C cells to mannose was eliminated by Zn^2+^ supplement (Fig. 3i). Altogether, our data demonstrate that Zn^2+^ concentration can affect enzyme activity of PMI and cellular response to mannose.

### ZIP10 enhances enzyme activity of PMI by increasing intracellular Zn^2+^ concentration

**Fig. 4:**
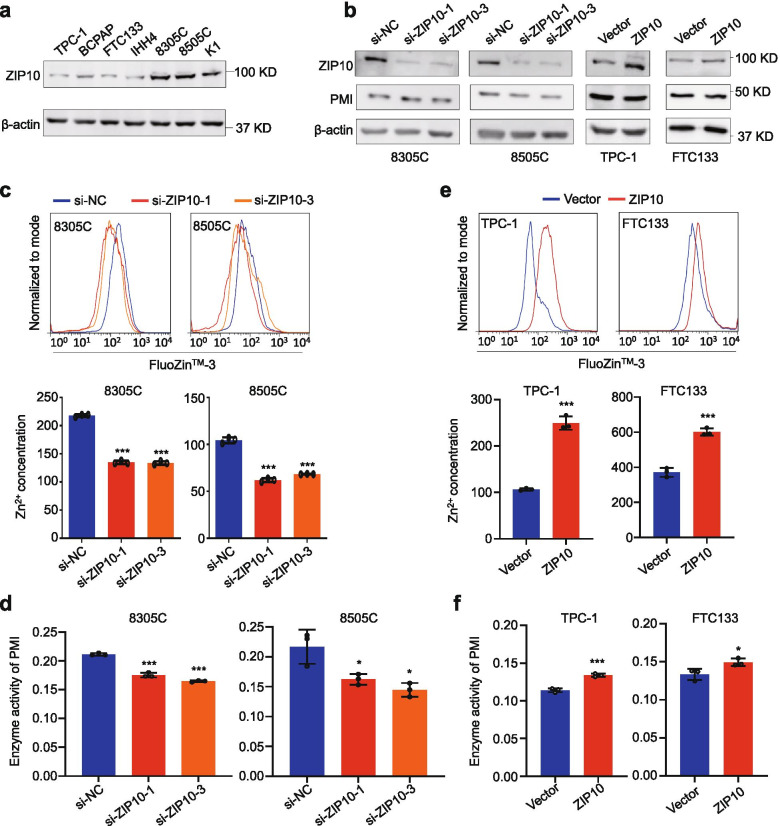
ZIP10 increases intracellular Zn^2+^ concentration and enzyme activity of PMI. **a** Western blot analysis of ZIP10 in thyroid cancer cell lines. β-actin was used as a loading control. **b** Western blot analysis showing ZIP10 knockdown in 8305C and 8505C cells by two different siRNAs (si-ZIP10–1 and si-ZIP10–3), and ectopic expression of ZIP10 in TPC-1 and FTC133 cells. β-actin was used as a loading control. **c** Measurement of intracellular Zn^2+^ concentration in ZIP10 knockdown-8305C/8505C cells and control cells by flow cytometer (upper panels). Statistical analysis of fluorescence intensity by Student’s t test (lower panels). **d** Measurement of enzyme activity of PMI in ZIP10 knockdown-8305C/8505C cells and control cells. **e** Measurement of intracellular Zn^2+^ concentration in ZIP10 overexpression-TPC-1/FTC133 and control cells by flow cytometer (upper panels). Statistical analysis of fluorescence intensity by Student’s t test (lower panels). **f** Measurement of enzyme activity of PMI in ZIP10 overexpression-TPC-1/FTC133 and control cells. The data were presented as mean ± SD. Statistically significant differences were indicated: *, *P* < 0.05; ***, *P* < 0.001

There is evidence showing that ZIP10, a Zn^2+^ transporter, transports zinc ions from extracellular area to the cytoplasm, thereby affecting cellular Zn^2+^ concentration [22]. Thus, we investigated ZIP10 expression in thyroid cancer cells by western blot analysis. We are surprised to find that ZIP10 expression in mannose-insensitive cell lines 8305C, 8505C and K1 was clearly elevated compared with mannose-sensitive cell lines TPC-1, BCPAP, FTC133 and IHH4 (Fig. 4a and Additional file 2: Fig. S6a). We next knocked down ZIP10 in 8305C and 8505C cells, and ectopically expressed ZIP10 in TPC-1 and FTC133 cells. The result showed that knockdown or overexpression of ZIP10 did not change PMI expression (Fig. 4b and Additional file 2: Fig. S6b and c). However, ZIP10 knockdown significantly decreased intracellular Zn^2+^ concentration (Fig. 4c) and enzyme activity of PMI (Fig. 4d), while ZIP10 overexpression increased Zn^2+^ concentration (Fig. 4e) and enzyme activity of PMI (Fig. 4f) compared with the control. These observations suggest that ZIP10 enhances enzyme activity of PMI by increasing intracellular Zn^2+^ concentration.

### ZIP10 regulates the response of mannose-insensitive thyroid cancer cells to mannose

To determine the role of ZIP10 in thyroid cancer, we first analyzed its expression using the Cancer Genome Atlas (TCGA) database, and found that *ZIP10* was significantly upregulated in thyroid cancers compared with control subjects (Additional file 2: Fig. S7a). Besides, we found that, compared with the control, ZIP10 knockdown significantly inhibited cell proliferation and colony formation in 8305C cells (Additional file 2: Fig. S7b-d), while ZIP10 overexpression promoted cell proliferation and colony formation in FTC133 cells (Additional file 2: Fig. S7e and f), indicating oncogenic role of ZIP10 in thyroid cancer.

**Fig. 5:**
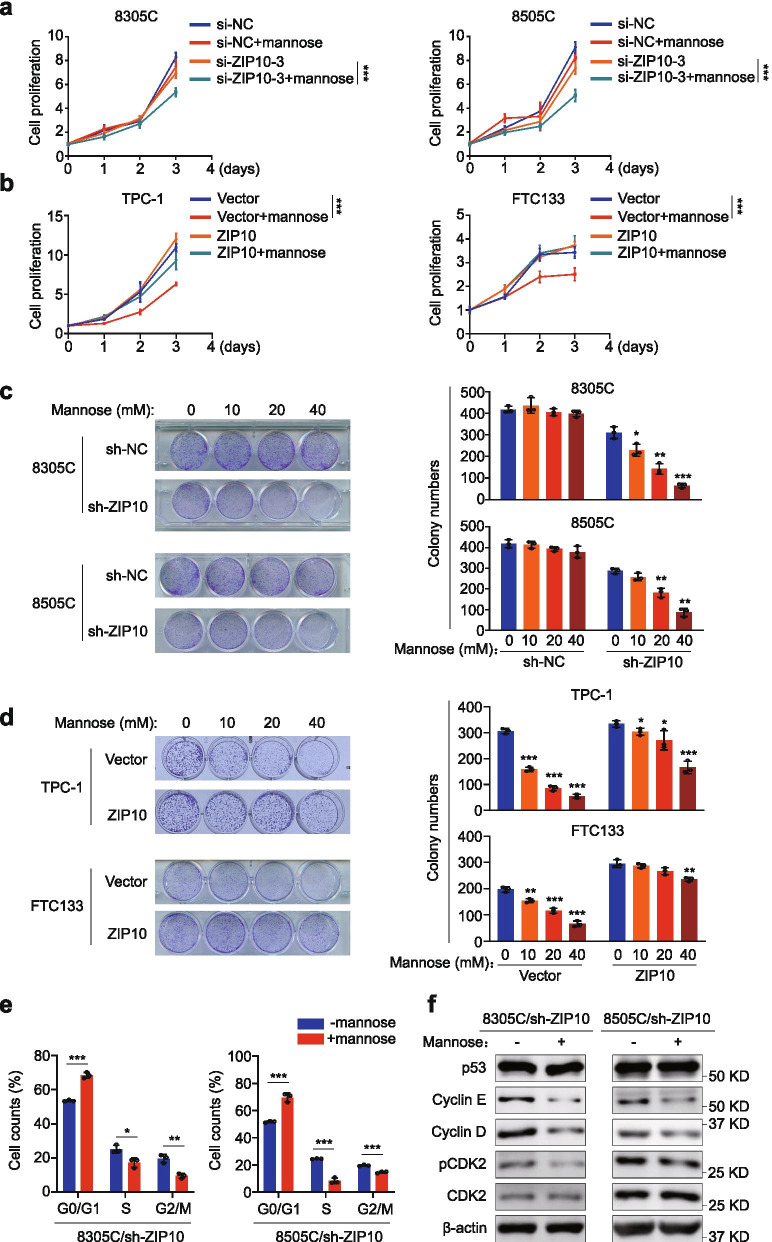
ZIP10 decreases thyroid cancer cell responsiveness to mannose. **a** The proliferation of ZIP10 knockdown-8305C/8505C cells and control cells treated with 20 mM mannose treatment or not. **b** The proliferation of ZIP10 overexpression-TPC-1/FTC133 cells and control cells treated with 20 mM mannose or not. **c** Colony formation of ZIP10 knockdown-8305C/8505C cells and control cells treated with mannose in a dose-dependent manner. **d** Colony formation of ZIP10 overexpression-TPC-1/FTC133 cells and control cells treated with mannose in a dose-dependent manner. The representative colony images were shown in the upper panels. Colony numbers were then counted and presented in the lower panels. **e** ZIP10 knockdown-8305C and 8505C cells were treated with mannose or not for 24 h, and stained with PI. Cell cycle distributions were then analyzed by flow cytometry. **f** Western blot analysis of cell cycle-related molecules (including p53, cyclin E and cyclin D, pCDK2 and CDK2) in ZIP10 knockdown-8305C and 8505C after a 24-h mannose treatment. β-actin was used as a loading control. The data were presented as mean ± SD. Statistically significant differences were indicated: *, *P* < 0.05; **, *P* < 0.01; ***, *P* < 0.001

To determine whether ZIP10 impairs the response of thyroid cancer cells to mannose, we knocked down ZIP10 in mannose-insensitive cell lines 8305C and 8505C, and tested its effect on cell proliferation. The results showed that ZIP10 knockdown dramatically increased the response of these cells to mannose compared to the control (Fig. 5a). Conversely, we ectopically expressed ZIP10 in mannose-sensitive cell lines TPC-1 and FTC133, and expectedly found that ZIP10 overexpression decreased the response of these cells to mannose compared with the control (Fig. 5b). Next, we found that mannose inhibited colony formation of ZIP10 knockdown-8305C and 8505C cells in a dose-dependent manner, while hardly changed that of control cells (Fig. 5c). Meanwhile, we also evaluated colony formation ability in ZIP10 overexpressed-TPC-1 and FTC133 cells, and found that, compared with ZIP10 overexpressed-cells, mannose more clearly inhibited colony formation of control cells in a dose-dependent manner (Fig. 5d), further supported the above conclusion.

We also evaluated cell cycle distributions in ZIP10 knockdown-8305C and 8505C cells after a 24-h mannose treatment, and found that mannose induced a significant G_0_/G_1_ phase arrest in these cells (Fig. 5e and Additional file 2: Fig. S8a). As supported, mannose clearly inhibited the expression of cyclin D, cyclin E and pCDK2 (Fig. 5f and Additional file 2: Fig. S8b). Taken together, our data indicate that ZIP10 decreases the response of thyroid cancer cells to mannose by enhancing enzyme activity of PMI.

### Mannose selectively inhibits the growth of thyroid cancer cells in vivo

**Fig. 6:**
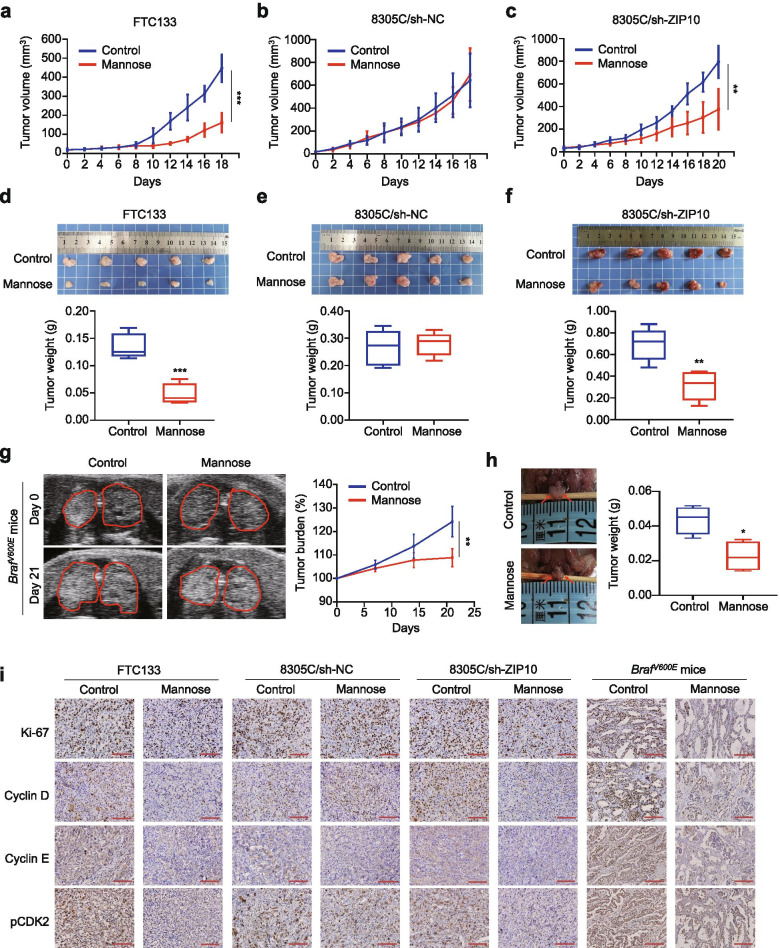
Selective in vivo anti-tumor activity of mannose. **a-c** FTC-133 and 8305C/sh-NC cell-derived xenograft tumors were treated with mannose (15% drinking water daily plus 20% mannose water oral gavage four times a week) or sterilized water (control) for 18 days when the tumor grew to 10–30 mm^3^. The xenografts derived from 8305C/sh-ZIP10 cells were similarly treated with mannose or sterilized water for 20 days when the tumor grew to 20–60 mm^3^. D_0_ is the beginning of mannose treatment. **d**-**f** Images of dissected tumors from the above groups (upper panels). The mean tumor weight in different groups was presented as box-whisker plot (lower panels). **g** *Braf*^*V600E*^ transgenic mice received 20% mannose water or sterilized water by oral gavage (200 μL) every day at week 4. Ultrasound examination of the thyroid was then performed weekly to evaluate tumor burden during treatment. Left panels indicate representative ultrasound images of thyroid in mannose-treated or control mice at day 0 and day 21. Image depth: 9 mm. Focus depth: 5 mm. Ultrasonic probe: MS550D. Tumor proliferation burden were then quantified and presented in the right panel. **h** Left panels showing the images of dissected tumors of mannose-treated or control mice. The mean tumor weight was measured and presented as box-whisker plot (right panel). **i**, IHC staining of Ki-67, cyclin D, cyclin E and pCDK2 in tumor tissues form the indicated groups. Scale bar: 100 μm. The data were presented as mean ± SD. Statistically significant differences were indicated: *, *P* < 0.05; **, *P* < 0.01; ***, *P* < 0.001

To determine in vivo anti-tumor activity of mannose, nude mice bearing xenograft tumors derived from FTC133 cells, ZIP10 knockdown-8305C cells (8305C/sh-ZIP10) and its control cells (8305C/sh-NC) were treated with mannose or sterilized water (Control) at the indicated time points. As shown in Fig. 6a, FTC-133 cell-derived xenograft tumors grew more slowly in the mannose-treated group relative to control group. Expectedly, mannose hardly changed the growth of 8305C/sh-NC cell-derived xenograft tumors (Fig. 6b), while significantly inhibited the growth of 8305C/sh-ZIP10 cell-derived xenograft tumors (Fig. 6c).

Considering that mannose might help lose weight [14], we thus measured body weight of mice during mannose treatment. The results demonstrated that body weight of mice was virtually unchanged by mannose (Additional file 2: Fig. S9a). One possible reason is the limited number of mannose treatment. At the end of the experiments, tumors were isolated and weighted. The results showed that mannose treatment significantly decreased the weight of FTC133 or 8305C/sh-ZIP10 cell-derived xenograft tumors compared with the control, while did not change that of 8305C/sh-NC cell-derived xenograft tumors (Fig. 6d-f). Meanwhile, we verified decreased expression of ZIP10 in 8305C/sh-ZIP10 cell-derived xenograft tumors compared with control tumors by western blot analysis; however, there was no statistically difference in PMI expression between these two groups (Additional file 2: Fig. S9b).

To further prove anti-tumor activity of mannose in primary thyroid cancers, we established a mouse model of *BRAF*^V600E^-derived papillary thyroid cancer (PTC) by crossing the *Braf*^CA^ mice with *TPO-Cre* mice. When the mice grew to 4 weeks, the thyroid became larger compared to normal mice, and the H&E staining of thyroid tissues showed the characteristics of PTC (Additional file 2: Fig. S9c and d). Next, we began to give 20% mannose or not daily to treat these mice at week 4 for 21 days. Meanwhile, ultrasound examination of the thyroid was performed weekly to assess tumor burden during mannose treatment. The results showed that mannose inhibited proliferation rate compared with the control (Fig. 6g). We weighted the isolated tumors at the end of experiments, and found that mannose significantly reduced tumor volume and weight compared to control (Fig. 6h).

Considering that Ki-67 is an important marker of tumor proliferation [35], we assessed the proportion of positive Ki-67 cells in xenograft tumors by IHC assay. Meanwhile, we also evaluated the expression of the above cell cycle-related molecules in tumor tissues by IHC analysis. The results showed that the levels of Ki-67, cyclin D, cyclin E and pCDK2 were clearly decreased upon mannose treatment in FTC-133, 8305C/sh-ZIP10 cell-derived xenograft tumors, and primary tumors of *BRAF*^V600E^ transgenic mice. However, mannose did not change the levels of these markers in 8305C/sh-NC cell-derived xenograft tumors (Fig. 6i and Additional file 2: Fig. S9e). These data further support the above conclusions that mannose selectively inhibited thyroid cancer cell growth, and ZIP10 is a negative determinant for its anti-tumor activity.

### Mannose selectively inhibits the glycolysis of thyroid cancer cells

**Fig. 7:**
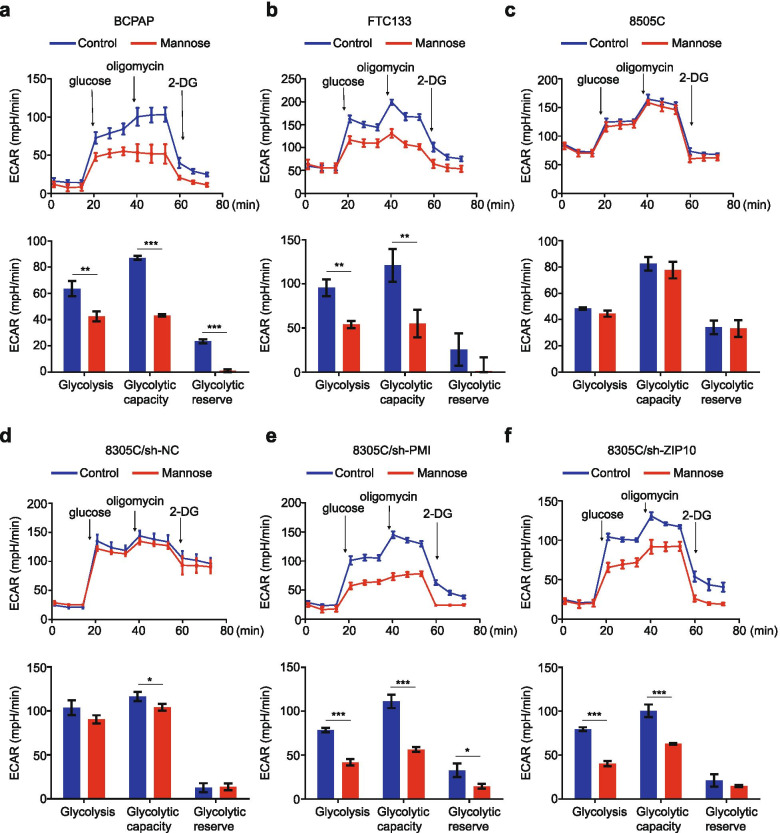
Selective inhibition of cellular glycolysis by mannose. **a**-**c** The indicated cells were treated with mannose or sterilized water (Control) for 36 h. The ECAR was measured three times at basal state, sequential injection of glucose, oligomycin and 2-deoxy-D-glucose (upper panels). The levels of glycolysis, glycolytic capacity and glycolytic reserve were calculated and presented in the lower panels. **d**-**f** PMI or ZIP10 knockdown-8305C cells and control cells were treated with mannose or sterilized water for 36 h. The ECAR was measured three times at basal state, sequential injection of glucose, oligomycin and 2-deoxy-D-glucose (upper panels). The levels of glycolysis, glycolytic capacity and glycolytic reserve were calculated and presented in the lower panels. The data were presented as mean ± SD. Statistically significant differences were indicated: *, *P* < 0.05; **, *P* < 0.01; ***, *P* < 0.001

In fact, mannose is catalyzed into mannose-6-phosphate (M-6-P), thereby inhibiting cellular glycolysis and subsequent tumor growth [36]. Thus, we performed Seahorse XF Glycolytic stress tests to determine whether mannose impairs the glycolysis of thyroid cancer cells after a 36-h treatment. Usually, the parameters of glycolysis, glycolytic capacity and glycolytic reserve represent for cellular glycolytic levels [37]. The results showed that glycolytic levels of mannose-sensitive cells, such as BCPAP and FTC133, significantly decreased after mannose treatment (Fig. 7a and b), while the glycolytic levels of mannose-insensitive cells, such as 8505C, maintained barely unchanged after mannose treatment (Fig. 7c).

As mentioned above, thyroid cancer cells with high enzyme activity of PMI or ZIP10 expression were insensitive to mannose. Thus, we speculate that, in mannose-insensitive thyroid cancer cells, M-6-P is immediately converted to fructose-6-phosphate, making it unable to accumulate and thus loss inhibitory effect on the glycolysis. To prove this, we performed Seahorse XF Glycolytic stress tests to determine the effect of mannose on the glycolysis of mannose-insensitive cell line 8305C when PMI or ZIP10 was knocked down. We expectedly found that, compared with the control (8305C/sh-NC; Fig. 7d), mannose clearly suppressed cellular glycolysis in PMI or ZIP10 knockdown 8305C cells (Fig. 7e and f). These results indicate that mannose selectively kills thyroid cancer cells by reducing cellular glycolysis.

**Fig. 8:**
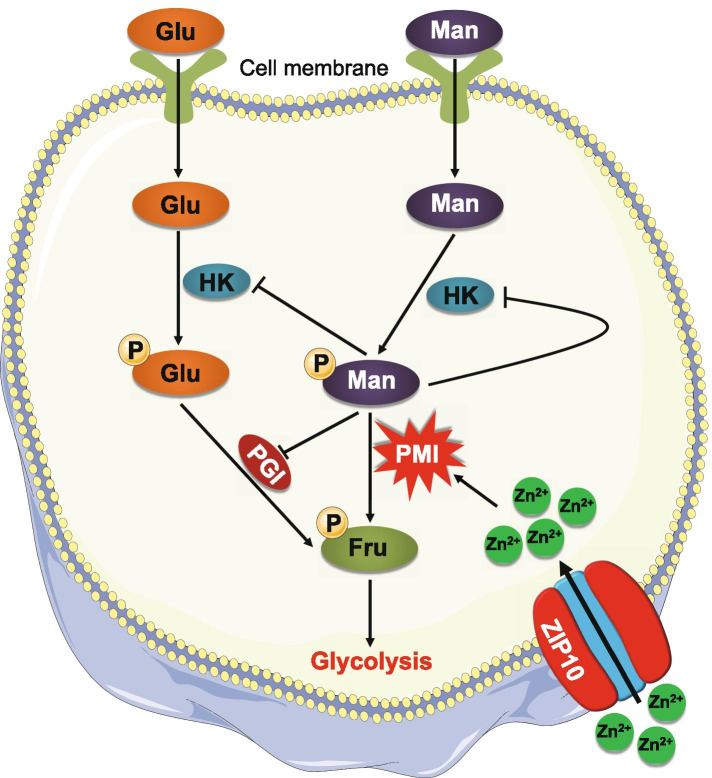
A schematic model of mannose killing thyroid cancer cells by inhibiting cellular glycolysis. Mannose enters the cell and converts into mannose-6-phophate which inhibits the enzymes of glycolytic pathway, such as hexokinase and phosphoglucose isomerase, thereby hindering cellular glycolysis. However, when PMI is highly activated by ZIP10-mediated extracellular Zn^2+^ influx, mannose-6-phosphate can be converted into fructose-6-phosphate by PMI, promoting cellular glycolysis and malignant phenotypes of cancer cells. Thus, ZIP10 is a negative determinant for the response of thyroid cancer cells to mannose

Summarizing the above findings, we propose a simple model to elucidate the mechanism of mannose selectively killing thyroid cancer cells (Fig. 8). Briefly, Mannose enters cell and converts into M-6-P, while M-6-P be further transforms into F-6-P under the catalysis of PMI. In this process, ZIP10 is a positive determinant for enzyme activity of PMI in thyroid cancer cells by transporting Zn^2+^ from extracellular space into cytoplasm. Thus, ZIP10 depletion enhances the inhibitory effect of mannose on cellular glycolysis by increasing M-6-P accumulation, thereby improving the response of mannose-insensitive thyroid cancer cells to mannose.

## Discussion

Some ingredients existing in natural foods have been proved to kill cancer cells as drugs, such as Vitamin C and curcumin [27, 38]. These compounds not only effectively suppress the growth of malignancies, but also exert no harmful effect on normal tissues. Mannose, which exists in various vegetables and fruits, selectively inhibits tumor progression. This selectivity is highly related to expression status of PMI that converts M-6-P to F-6-P. When PMI expression is extremely low, M-6-P cannot convert into F-6-P and accumulates in tumor cells, thereby killing cancer cells by inhibiting cellular glycolysis [16]. However, its role in thyroid cancer still remains elusive.

In the present study, we demonstrated that mannose selectively killed thyroid cancer cells, which was similar to a previous study [16]. Surprisingly, we did not find that there was statistical difference in PMI expression between mannose-sensitive cells and mannose-insensitive cells. However, mannose-insensitive cells became sensitive to mannose when PMI was knocked down in these cells. There was study showing that enzyme activity of PMI dictates the response of LPS-activated macrophage to mannose [39]. Thus, we tested enzyme activity of PMI in a panel of thyroid cancer cells, and found that enzyme activity of PMI was higher in mannose-insensitive cells than mannose-sensitive cells, indicating that high enzyme activity of PMI impairs the response of thyroid cancer cells to mannose.

It has been disclosed that Zn^2+^ plays an essential role in maintaining enzyme activity of PMI by binding to its catalytic core area [17, 18]. Meanwhile, after extracting PMI proteins in vitro and chelating Zn^2+^ with EDTA, its catalytic activity significantly reduced [26]. Previous study implied that Zn^2+^ did not change the structure of PMI protein [40]. Thus, we attempted to determine whether there was a similar mechanism existing in thyroid cancer cells. Our results demonstrated that Zn^2+^ chelator TPEN treatment significantly reduced intracellular Zn^2+^ concentration and inhibited enzyme activity of PMI, while did not change PMI expression. Besides, we expectedly found that TPEN treatment obviously improved the response of mannose-insensitive thyroid cancer cells to mannose, and Zn^2+^ supplement could effectively reverse this effect. It is well-known that zinc transporter ZIP10 is required for the entry of Zn^2+^ into the cell [41]. Next, we investigated ZIP10 expression in thyroid cancer cells, and found that ZIP10 expression was clearly higher in mannose-insensitive cells than mannose-sensitive cells. Functional studies demonstrated oncogenic role of ZIP10 in thyroid cancer, which was consistent with a previous study showing that ZIP10 promoted zinc-triggered mitosis [42]. Importantly, ZIP10 knockdown decreased intracellular Zn^2+^ concentration and enzyme activity of PMI, enhancing the response of mannose-insensitive cells to mannose. Conversely, ectopic expression of ZIP10 in mannose-sensitive cells decrease their response to mannose. These findings indicate that expression status of ZIP10 is a major determinant for anti-tumor activity of mannose in thyroid cancer cells, and may be a potential therapeutic target to sensitize the response of cancer cells to mannose.

Warburg effect is a special metabolism found in cancer cells which tend to obtain energy through anaerobic respiration in spite of sufficient oxygen, thus the inhibition of cellular glycolysis will be an effective strategy for cancer therapy [43, 44]. M-6-P has been demonstrated to impede cellular glycolysis by suppressing hexokinase, phosphoglucose isomerase and glucose-6-phosphate dehydrogenase [36]. Thus, it is reasonable to speculate that mannose kills thyroid cancer cells by this mechanism. When mannose enters into the cell, it transforms into M-6-P which accumulates when PMI enzyme activity is low, thereby decreasing cellular glycolytic levels [16, 45]. As supported, our data showed that mannose treatment significantly suppressed the glycolysis of mannose-sensitive cells, while did not change that of mannose-insensitive cells. However, PMI or ZIP10 knockdown in mannose-insensitive cells could effectively inhibit enzyme activity of PMI, thereby promoting the inhibitory effect of mannose on cellular glycolysis.

Evidently, mannose can enhance the response of cancer cells to chemotherapy due to the accumulation of mannose-6-phosphate and subsequent inhibition of glycolysis [16]. Besides, a previous study showed that PKM2 promoted chemotherapy resistance by enhancing the glycolysis in ER-positive breast cancer [46]. However, whether mannose can improve the response of thyroid cancer cells to chemotherapy needs to be explored in the near further. In addition to chemotherapy, the radiotherapy is another therapeutic strategy for thyroid cancer. There is evidence indicating that targeting HIF1 enhanced the radiosensitivity of breast cancer cells by reducing cellular glycolytic levels and the content of lactate acid [47], suggesting that mannose may improve the response of thyroid cancer cells to ionizing radiation by inhibiting cellular glycolysis.

In recent years, immunotherapy has become a highly promising strategy for cancer treatment [48]. There is increasing evidence hinting that inhibition of glycolysis could effectively improve the response of cancer cells to CTLA-4 blocker by impairing the stability of Treg cells [49]. Besides, lactic acid, the production of glycolysis, has been proved to facilitate the infiltration of Treg cells in tumor, interrupting the lethal function of effector T cells [50]. Thus, we speculate that mannose may improve the efficacy of immune checkpoint inhibitors by suppressing cellular glycolysis. These observations support that mannose has potential clinical use in thyroid cancer therapy when combined with chemotherapy, radiotherapy or immunotherapy.

## Conclusion

In summary, by a series of in vitro and in vivo experiments, we demonstrate that mannose selectively kills thyroid cancer cells, and this effect is highly dependent on enzyme activity of PMI rather than its expression. Further studies reveal that PMI can be activated by zinc transport protein ZIP10 through promoting Zn^2+^ influx, thereby decreasing the response of thyroid cancer cells to mannose. Thus, our data highlight a crucial role of expression status of ZIP10 in affecting the response of thyroid cancer cells to mannose, and offer a mechanistic rationale for exploring clinical use of mannose in thyroid cancer therapy, especially combining with chemotherapy, radiotherapy or immunotherapy.

## Supplementary Information

**Additional file 1: Table S1.** The STR DNA profiling of cell lines used in this study. **Table S2**. Short interfering RNAs (siRNAs) used in this study. **Table S3**. Lentivirus short hairpin RNAs (sh-RNAs) used in this study. **Table S4**. The primer sequences used in this study.

**Additional file 2: Figure S1.** The effect of mannose on the proliferation of immortalized thyroid cell line Hthy-ori3–1. **Figure S2.** The effect of mannose on cell cycle distributions in thyroid cancer cells. **Figure S3.** The effect of PMI knockdown on cell cycle distributions in thyroid cancer cells. **Figure S4.** The effect of PMI knockdown on its enzyme activity. **Figure S5.** Zn^2+^ chelator TPEN increases the response of mannose-insensitive cell lines K1 and 8505C to mannose. **Figure S6**. Distinct expression of ZIP10 in thyroid cancer cell lines. **Figure S7.** Oncogenic role of ZIP10 in thyroid cancer cells. **Figure S8.** The effect of mannose on cell cycle distributions in ZIP10 knockdown-thyroid cancer cells. **Figure S9.** In vivo anti-tumor effect of mannose.

## Data Availability

All data generated or analyzed during this study are included in this article.

## Abbreviations

PMI: Phosphate mannose isomerase
Zn^2+^: Zinc ions
ZIP10: Zinc transporter 10
M-6-P: Mannose-6-phosphate
F-6-P: Mannose-6-phosphate
GLUT: Glucose transporter
MOI: Multiplicity of infection
qRT-PCR: Quantitative real time polymerase chain reaction
ECAR: Extracellular acid rate
