# Supplementary material for: ZIP10 is a negative determinant for anti-tumor effect of mannose in thyroid cancer by activating phosphate mannose isomerase
Source: J Exp Clin Cancer Res. 2021 Dec 9;40:387. doi: 10.1186/s13046-021-02195-z (PMC8656095; doi:10.1186/s13046-021-02195-z)
Supplement: Supplementary file 1 — Additional file 1: Table S1. The STR DNA profiling of cell lines used in this study. Table S2. Short interfering RNAs (siRNAs) used in this study. Table S3. Lentivirus short hairpin RNAs (sh-RNAs) used in this study. Table S4. The primer sequences used in this study. [file 13046_2021_2195_MOESM1_ESM.docx]

**Supplementary Tables**

**Table S1**. The STR DNA profiling of cell lines used in this study

| Cell lines | Amelo | D13S317 | D18S51 | D21S11 | D3S1358 | D5S818 | D7S820 | D8S1179 | FGA | vWA | CSF1PO | D16S539 | TH01 | TPOX |
| --- | --- | --- | --- | --- | --- | --- | --- | --- | --- | --- | --- | --- | --- | --- |
| K1 | X/Y | 11/14 | 18/18 | 30/31.2 | 18/18 | 10/11 | 11/11 | 15/15 | 21/24 | 17/18 |  |  |  |  |
| BCPAP | X/X | 12/12 | 13/17 | 30/31.2 | 16/17 | 10/11 | 10/10 | 12/13 | 20/23 | 14/17 |  |  |  |  |
| FTC133 | X/X | 11/11 | 11/12 | 32.2/32.2 | 15/15 | 12/12 | 9/10 | 10/10 | 21/21 | 15/18 |  |  |  |  |
| TPC-1 | X/X | 11/12 | 13/16 | 30/31.2 | 16/17 | 8/10 | 11/11 | 11/17 | 20/21 | 14/18 |  |  |  |  |
| 8305C | X/X | 9/9 |  |  |  | 10/13 | 8/10 |  |  | 14/16 | 9/12 | 10/11 | 6/7 | 8/8 |
| IHH4 | X/Y | 10/13 |  |  |  | 9/13 | 9/10 |  |  | 16/18 | 12/12 | 9/11 | 9/9 | 11/11 |
| 8505C | X/X | 13/13 |  |  |  | 10/11 | 10/10 |  |  | 17/19 | 12/13 | 12/12 | 6/9 | 11/11 |

Dashed lines indicate that these loci were not analyzed in this study.

**Table S2**. Short interfering RNAs (siRNAs) used in this study

| siRNAs Sense (5’-3’) Antisense (5’-3’) |
| --- |
| si-PMI-1 GGCUCAAAGGUCAAGGACATT UGUCCUUGACCUUUGAGCCTT |
| si-PMI-3 GGAGAUUGUAACCUUUCUATT UAGAAAGGUUACAAUCUCCTT |
| si-ZIP10-1 CCACAAACCUGAUCGUGUATT UACACGAUCAGGUUUGUGGTT |
| si-ZIP10-3 ACAGCAUCGUGGAAUGACATT UGUCAUUCCACGAUGCUGUTT |

**Table S3**. Lentivirus short hairpin RNAs (sh-RNAs) used in this study

| shRNAs | Top Strand | Bottom Strand |
| --- | --- | --- |
| sh-PMI | GATCCGGCTCAAAGGTCAAG  GACATTCAAGAGATGTCCTT  GACCTTTGAGCCTTTTTTG | AATTCAAAAAAGGCTCAAAG  GTCAAGGACATCTCTTGAATG  TCCTTGACCTTTGAGCCG |
| sh-ZIP10 | GATCCGACAGCATCGTGGAAT  GACATTCAAGAGATGTCATTC  CACGATGCTGTTTTTTTG | AATTCAAAAAAACAGCATCGT  GGAATGACATCTCTTGAATGTC  ATTCCACGATGCTGTCG |

**Table S4**. The primer sequences used in this study

| Genes Forward primer (5’-3’) Reverse Primer (5’-3’) |
| --- |
| *PMI*  GACAGGCTCTTTCTCCCAACAC CAGAGTCCAGTGCCAAGACCTT |
| *ZIP10*  AACCTGGTTCCTGAAGATGAGGC GATCACGCCTAGCAAGGAAAGC |
| *18S* CGCCGCTAGAGGTGAAATTC CTTTCGCTCTGGTCCGTCTT |
