# Supplementary material for: ZIP10 is a negative determinant for anti-tumor effect of mannose in thyroid cancer by activating phosphate mannose isomerase
Source: J Exp Clin Cancer Res. 2021 Dec 9;40:387. doi: 10.1186/s13046-021-02195-z (PMC8656095; doi:10.1186/s13046-021-02195-z)
Supplement: Supplementary file 2 — Additional file 2: Figure S1. The effect of mannose on the proliferation of immortalized thyroid cell line Hthy-ori3–1. Figure S2. The effect of mannose on cell cycle distributions in thyroid cancer cells. Figure S3. The effect of PMI knockdown on cell cycle distributions in thyroid cancer cells. Figure S4. The effect of PMI knockdown on its enzyme activity. Figure S5. Zn2+ chelator TPEN increases the response of mannose-insensitive cell lines K1 and 8505C to mannose. Figure S6. Distinct expression of ZIP10 in thyroid cancer cell lines. Figure S7. Oncogenic role of ZIP10 in thyroid cancer cells. Figure S8. The effect of mannose on cell cycle distributions in ZIP10 knockdown-thyroid cancer cells. Figure S9. In vivo anti-tumor effect of mannose. [file 13046_2021_2195_MOESM2_ESM.docx]

**Supplementary Figures**

Fig. S1. The effect of **mannose** on the proliferation of **immortalized thyroid cell line Hthy-ori3-1**. Hthy-ori3-1 cells were treated with mannose at the concentration of 20 mM or sterilized water (control) for 3 days. The MTT assay was then performed to measure cell proliferation.

Fig. S2. The effect of **mannose** on **cell cycle distributions in thyroid cancer cells**. **a**, The representative images of cell cycle distributions in BCPAP, FTC133, 8305C and 8505C cells treated with 20 mM or sterilized water (control) for 24 h (related to Fig. 1d). **b**, The densitometry analysis was performed to measure the levels of p53, cyclin E, cyclin D, pCDK2 and CDK2 proteins in the indicated cells (related to Fig. 1e). The data were presented as mean ± SD. *, *P* <0.05; **, *P* <0.01.

Fig. S3. The effect of PMI knockdown on **cell cycle distributions in thyroid cancer cells**. **a**, The densitometry analysis was performed to measure the levels of PMI proteins in a panel of thyroid cancer cell lines (related to Fig. 2a). **b**, The densitometry analysis was performed to measure the levels of PMI proteins in 8305C, 8505C and K1 cells (related to Fig. 2b). **c**, Western blot analysis showing PMI knockdown in 8305C, 8505C and K1 cells by lentivirus system. β-actin was used as a loading control. **d**, The representative pictures for cell cycle distributions of PMI knockdown- 8305C/8505C cells (related to Fig. 2e). **e**, The densitometry analysis was performed to measure the levels of p53, cyclin E, cyclin D, pCDK2 and CDK2 proteins in PMI knockdown- 8305C/8505C cells with the indicated treatments (related to Fig. 2f). The data were presented as mean ± SD. **, *P* <0.01; ***, *P* <0.001.

Fig. S4. The effect of PMI knockdown on its enzyme activity. Enzyme activity of PMI in PMI knockdown-8305C/8505C cells and control cells was examined by cysteine carbazole sulfuric acid method. The O.D. values were measured in a spectrophotometer at 560 nm. The data were presented as mean ± SD. **, *P* <0.01.

Fig. S5. Zn^2+^ chelator TPEN increases the response of mannose-insensitive cell lines **K1 and 8505C to** mannose. Colony formation assay was performed in K1 and 8505C cells with the indicated treatments. The representative colony images were shown in the upper panels. Colony numbers were then counted and presented in the lower panels. The data were presented as mean ± SD. **, *P* <0.01.

Fig. S6. **Distinct expression of** ZIP10 in thyroid cancer cell lines. **a**, The densitometry analysis was performed to measure the levels of ZIP10 proteins in a panel of thyroid cancer cell lines (related to Fig. 4a). **b**, The densitometry analysis was performed to measure the levels of ZIP10 and PMI proteins in ZIP10 kncockdown-8305C/8505C cells and control cells (related to Fig. 4b). **c**, The densitometry analysis was performed to measure the levels of ZIP10 and PMI proteins in ZIP10 overexpression-TPC-1/FTC133 cells and control cells (related to Fig. 4b). The data were presented as mean ± SD. *, *P* <0.05; **, *P* <0.01; ***, *P* <0.001.

Fig. S7. **Oncogenic role of** ZIP10 in thyroid cancer cells. **a**, Relative mRNA expression of ZIP10 in thyroid cancers (T) and normal thyroid tissues (N) or matched normal thyroid tissues (MN) (data from TCGA database). **b**, Western blot analysis showing ZIP10 knockdown in 8305C and 8505C cells by lentivirus system. β-actin was used as a loading control. c, The proliferation of ZIP10 knockdown-8305C cells and control cells. d, Colony formation of ZIP10 knockdown-8305C cells and control cells. The representative colony images were shown in the left panels. Colony numbers were then counted and presented in the right panel. **e**, The proliferation of ZIP10 overexpression-FTC133 cells and control cells. f, Colony formation of ZIP10 overexpression-FTC133 cells and control cells. The representative colony images were shown in the left panels. Colony numbers were then counted and presented in the right panel. The data were presented as mean ± SD. *, *P* <0.05; **, *P* <0.01.

Fig. S8. The effect of **mannose** on **cell cycle distributions in ZIP10 knockdown-thyroid cancer cells**. **a**, The representative images of cell cycle distributions in ZIP10 knockdown-8305C /8505C cells after these cells were treated with 20 mM or sterilized water (control) for 24 h (related to Fig. 5e). **b**, The densitometry analysis was performed to measure the levels of p53, cyclin E, cyclin D, pCDK2 and CDK2 proteins in ZIP10 knockdown-8305C /8505C cells with the indicated treatments (related to Fig. 5f). The data were presented as mean ± SD. *, *P* <0.05; **, *P* <0.01; ***, *P* <0.001.

Fig. S9. *In vivo* anti-tumor effect of mannose. **a**, Growth curves for body weight of the indicated mice during mannose treatment. **b**, Western blot analysis of ZIP10 and PMI in the indicated xenograft tumors (left panel). GAPDH was used as a loading control. Corresponding statistical results were presented in right panels. **c**, The size of thyroid in *Braf^V600E^* mice and control mice at 4 weeks. **d,** H&E staining of thyroid in *Braf^V600E^* mice and control mice at 4 weeks. **e,** Quantitative analysis of the indicated proteins in tumor tissues from the indicated mice (related to Fig. 6i). The data were presented as mean ± SD. *, *P* <0.05; **, *P* <0.01; ***, *P* <0.001.
